# Emerging Trends of Nanotechnology and Genetic Engineering in Cyanobacteria to Optimize Production for Future Applications

**DOI:** 10.3390/life12122013

**Published:** 2022-12-02

**Authors:** Rajakumar Govindasamy, Ekambaram Gayathiri, Sathish Sankar, Baskar Venkidasamy, Palanisamy Prakash, Kaliaperumal Rekha, Varsha Savaner, Abirami Pari, Natesan Thirumalaivasan, Muthu Thiruvengadam

**Affiliations:** 1 Department of Orthodontics, Saveetha Dental College and Hospital, Saveetha Institute of Medical and Technical Sciences, Chennai 600077, India; 2 Department of Plant Biology and Plant Biotechnology, Guru Nanak College (Autonomous), Chennai 600042, India; 3 Department of Microbiology, Saveetha Dental College and Hospital, Saveetha Institute of Medical and Technical Sciences, Chennai 600077, India; 4 Department of Oral and Maxillofacial Surgery, Saveetha Dental College and Hospital, Saveetha Institute of Medical and Technical Sciences, Chennai 600077, India; 5 Department of Botany, Periyar University, Periyar Palkalai Nagar, Salem 636011, India; 6 Department of Environmental and Herbal Science, Tamil University, Thanjavur 613005, India; 7 Institute of Biological Science, SAGE University, Indore 452020, India; 8 Department of Botany, Seethalakshmi Achi College for Women, Pallathur, Sivaganga 630107, India; 9 Department of Periodontics, Saveetha Dental College and Hospital, Saveetha Institute of Medical and Technical Sciences, Chennai 600077, India; 10 Department of Applied Bioscience, College of Life and Environmental Sciences, Konkuk University, Seoul 05029, Republic of Korea

**Keywords:** cyanobacteria, nanomaterials, genetic engineering, environmental impacts, agriculture practices, biomedicals, biofuels

## Abstract

Nanotechnology has the potential to revolutionize various fields of research and development. Multiple nanoparticles employed in a nanotechnology process are the magic elixir that provides unique features that are not present in the component’s natural form. In the framework of contemporary research, it is inappropriate to synthesize microparticles employing procedures that include noxious elements. For this reason, scientists are investigating safer ways to produce genetically improved Cyanobacteria, which has many novel features and acts as a potential candidate for nanoparticle synthesis. In recent decades, cyanobacteria have garnered significant interest due to their prospective nanotechnological uses. This review will outline the applications of genetically engineered cyanobacteria in the field of nanotechnology and discuss its challenges and future potential. The evolution of cyanobacterial strains by genetic engineering is subsequently outlined. Furthermore, the recombination approaches that may be used to increase the industrial potential of cyanobacteria are discussed. This review provides an overview of the research undertaken to increase the commercial avenues of cyanobacteria and attempts to explain prospective topics for future research.

## 1. Introduction

**Figure 1:**
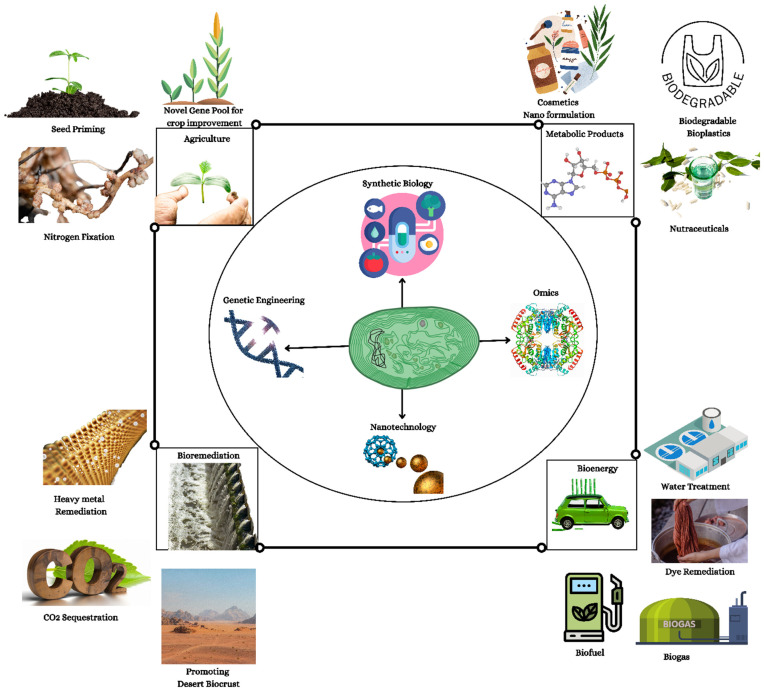
Cyanobacteria: A precious resource for a sustainable ecosystem.

The cyanobacteria (also termed blue-green algae) are the most numerous, diversified, and widely dispersed class of photosynthetic prokaryotes on the earth. Despite lacking a systematized chloroplast, its photosynthetic machinery is very comparable at the functional, structural, and molecular levels to that of higher plants and algae, with the notable distinction of their light absorption system that is made up of phycobilin chromophores. They belong to a particular phylum of bacteria known for their oxygenic photosynthesis. They are found all over the world in photic conditions. They are the most diversified phyla of prokaryotes that are primitive unicellular organisms that reproduce through binary fission and fragmentation. Some filamentous forms are completely multicellular, but others may branch out because of cell specialization and physiological competency between vegetative cells and heterocysts [1]. These species can survive in a wide range of habitats, including freshwater, marine, terrestrial, and harsh conditions like hot springs, the arctic, and the antarctic. They may also create symbiotic relationships with a range of hosts, including plants, algae, fungi, protists, and animals, as well as looser interactions with plants, growing epiphytically on tree bark and leaves. The development of these associations is under selection pressure due to the cyanobacterium’s capacity to fix nitrogen for the growth of the plant partner. Symbiotic relationships between plants and cyanobacteria are supported by the plant providing a unique habitat with certain physicochemical conditions that serve to shield and nourish the cyanobacteria [2]. Cyanobacteria are the only prokaryotes in which a circadian rhythm is evident. In contrast to heterotrophic microbes, cyanobacteria may utilize solar energies to convert CO_2_ into simple chemicals without the intermediate sugar moiety. The development of cyanobacteria occurs much more rapidly than that of microalgae and plants. Moreover, cyanobacteria are far more amenable to genetic manipulation. Complete genome sequencing of a photosynthetic organism began with the model *Synechocystis* sp. PCC 6803. Approximately 200 cyanobacteria genomes have been sequenced [3]. Thus, cyanobacteria have long been the topic of research in a variety of domains. Cyanobacterial bioengineering applications have received significant interest over the years. Researchers analyze the limitations and potential prospects of cyanobacterial genetic engineering and industrial and medicinal uses of cyanobacteria. In cyanobacteria, bioactive compounds with antiviral, antibacterial, antifungal, and anticancer properties have been identified. Cyanobacteria store polyhydroxyalkanoates that might replace petroleum-based plastics. Recently, abundant cyanobacterial consortiums capable of degrading oil components have been discovered in oil-polluted regions. The cyanobacteria within these consortiums helped in the degradation processes by providing oxygen, organic compounds, and fixed nitrogen to the associated oil-degrading bacteria. Hydrogen from cyanobacteria is a new renewable energy alternative to the now commercially available sources. Aquaculture, treatment of wastewater, agriculture, fertilizer, drug production, nutrient, enzyme production, and food processing all employ cyanobacteria. Therefore, novel strains of cyanobacteria that generate high-value products should be identified, and economically important strains should be genetically modified to improve the production of vital products. It is necessary to create metagenomic libraries to find novel genes that contribute to the production of biotechnologically pivotal components. In order to increase productivity on a vast scale, it is required to optimize cyanobacterial incubation conditions [4] (Figure 1).

The relevance of species interactions involving cyanobacteria is being more recognized, although the molecular intricacies of such interactions are still poorly understood. It is challenging to disentangle the benefits of co-occurring heterotrophic bacteria since most molecular investigations have been conducted in axenic laboratory cultures. For this reason, researchers have considered the development of laboratory models to examine these interactions. Important cyanobacterial species that can be genetically engineered have been understudied despite their potential importance. Modern gene-editing techniques and multi-omics approaches provide promising new insights into methods of acquiring genetic material from cyanobacteria, which have low-availability sources of genetic material. Combining information from the domains of biology, geochemistry, and ecology will provide a good understanding at both the ecological, molecular, and cellular levels [5]. Studies on the genomic, physiological, and ecological variety of cyanobacteria are important to explore their potential applications. It has a simple genome and needs minimal nutrients to grow. Numerous bioactive chemicals are known to be produced by cyanobacteria. The rapid multiplication of cyanobacteria widens several possibilities for their usage in industries as diverse as bioenergy production, nanotechnology, nutraceutical, and waste recycling.

## 2. Molecular Genetics of Cyanobacteria

Cyanobacteria function as prototype organisms for resolving scientific problems that are challenging to solve in angiosperms, and they may be desired species for investigation, which is not immediately connected to photosynthesis. Concerns about the photosynthetic system and its function, carbon fixation, light-regulated gene regulation, cellular differentiation, and stress adaptability in cyanobacteria might be addressed by applying molecular genetics. The genetic analysis and engineering of cyanobacteria require a natural or artificial DNA-absorbing mechanism. Thus, a mechanism of cellular competency may be used to absorb foreign DNA, even without recipient cell pretreatment. Most DNA-transformable cyanobacterial strains have inherent DNA absorption capability. Although the exact mechanism behind such a procedure is still unclear, it has been widely employed to genetically modify cyanobacterial cells either chromosomally or by using plasmids.

By recombining and internalizing donor chromosome DNA with homologous DNA in the recipient cell, chromosomal alteration is accomplished. It is possible to create and rapidly identify mutants based on their phenotype or a genetic marker by using donor DNA that has undergone either in vivo mutation or recombinant DNA changes in vitro before the transformation. This makes it possible to analyze the structure, function, and organization of genes by modifying the cyanobacterial genomes to cause insertion or deletion mutations. Plasmid transformation requires the introduction of a plasmid DNA capable of replication in the recipient cell. This allows for the construction of plasmid vectors and the construction of partial diploids or meroploids of cyanobacteria, each of which may be used to introduce foreign genes into the recipient cell and investigate their function. Cyanobacteria may also be transformed by chromosomally homologous non-replicating plasmids. Homologous recombination between the plasmid and the chromosome is possible; however, if a single recombination process happens between the homologous regions, the whole plasmid might be integrated into the cyanobacterial chromosome. Heterogeneous chromosomal architectures may be created from such mutations. So far, only unicellular strains are naturally capable of absorbing DNA. Conjugal DNA mediated transfer, which was developed for filamentous cyanobacteria, allows genetic material to be delivered into cells that were previously resistant to genetic transformation. The shuttle vectors used for this kind of transformation may be used to introduce genetic modifications into both the recipient cyanobacterium and Escherichia coli. The use of cyanophages as vectors is an alternative strategy for transferring DNA into cyanobacteria. Even though several cyanophages have been identified, no proof of gene transduction into cyanobacteria has been found. Our present knowledge of the ecology, physiology, and molecular genetics of cyanobacteria allows us to investigate the use of cyanobacteria for diverse biotechnological applications, such as the synthesis of certain allelochemicals, pesticides, and herbicides.

## 3. Cyanobacteria: Revolutionary Advances in Genome Engineering

**Figure 2:**
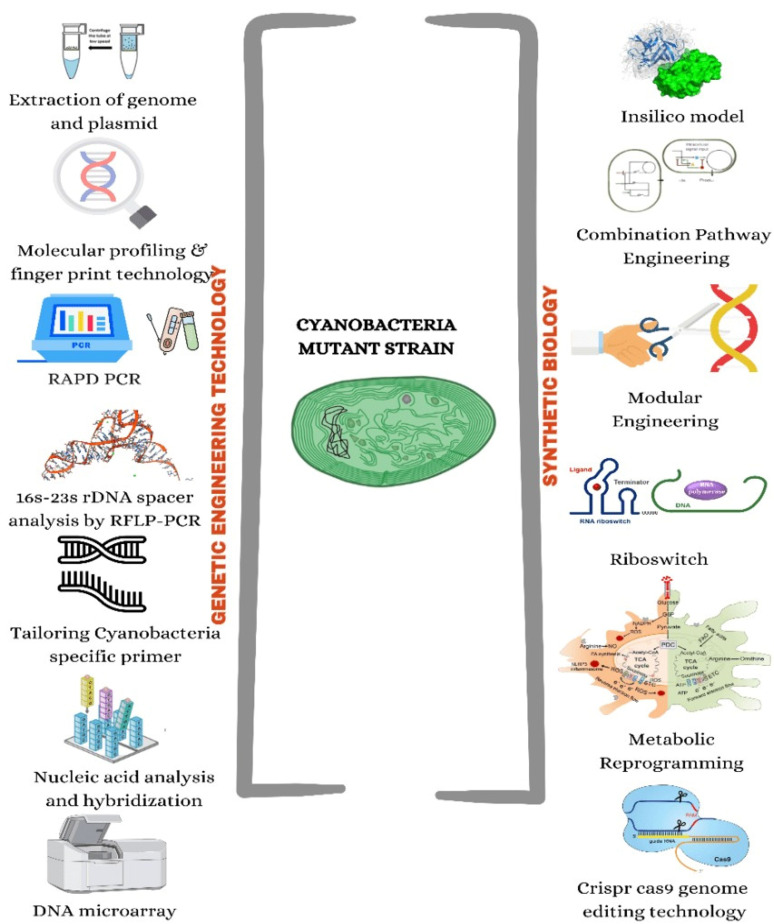
Genetic engineering technology and synthetic biology tools in cyanobacteria.

A wide variety of cyanobacterial morphologies, physiologies, and metabolic strategies have evolved throughout time as these organisms have adapted to harsh environments [6]. The only prokaryote known to adjust its metabolism according to time is cyanobacteria [7]. Recent cyanobacterial genome sequencing studies have revolutionized cyanobacterial research. One promising approach to increase strain fitness and heterologous production involves strategic host engineering using systems biology. In systems biology, engineered biological systems are created quickly, predictably, and easily, assembling and disassembling synthetic and native components. Recently, attempts have been made to develop the cyanobacterial systems biology toolkit, which consists of a variety of native and recombinant translational and transcriptional bio-parts, genomic editing and remodeling methods, and machine learning algorithms (Figure 2) [8].

The best reliable technique for engineering cyanobacteria is cis-genetic alteration via genome engineering since many cyanobacterial strains are susceptible to conformational change and molecular and cellular modifications for genetic mutation, insertion, or deletion. Generally, cyanobacteria strains are modified using a plasmid carrying the desired gene, marker gene, and flanking regions that are similar to the desired genomic loci [5]. Two popular model cyanobacteria, namely, *Synechocystis* sp. PCC 6803 and *Synechococcus elongatus* PCC 7942 have been transformed using this technique, which permits targeted chromosomal alterations to express heterologous genes and synthetic biosynthetic pathways [6]. Cyanobacteria are indeed a diverse class of microorganisms with a natural CRISPR system that has received very little research. The CRISPR-Cas cascade was discovered in 86 genomes, except for a phylogenetic subclade that comprised *Prochlorococcus* and marine *Synechococcus* [7]. The form, quantity, and proportion of CRISPR and Cas modules may vary [8]. *Coleofasciculus chthonoplastes* PCC 7420 was found to have the highest number of confirmed CRISPR loci in a cyanobacterial genome [9]. An examination of the genomes of cyanobacteria showed that the Cas proteins of Class 2 were less prevalent than those of Class 1. In Cyanobacteria, types I and III of the Class I CRISPR system and type V of the Class 2 effector Cas protein were discovered. When comparing unicellular and filamentous cyanobacteria, it has been shown that the former are more likely to contain the rare class of CRISPR, Class 2, in their genomes [8]. A diazotrophic unicellular cyanobacterium, *Cyanothece* sp. PCC 8801 possesses both Class 1 and 2 CRISPR loci in its genome. *Synechocystis* sp. PCC 6803, PCC 6714, *M. aeruginosa*, *Cyanothece* species PCC 8801, and *Anabaena* species PCC 7120 are the most studied strains for their natural CRISPR system. *Anabaena* sp. PCC 7120 comprises 3 class 1, one class 2, and five linkers, whereas *Synechocystis* sp. PCC 6803 has 3 CRISPR-Cascade sets in plasmids [8,10].

## 4. Enhanced Conversion of Photoenergy by Genetically Engineered Cyanobacteria

Microorganisms, as part of their metabolism, transport electrons to external electron acceptors are termed exoelectrogens. Microbial fuel cells (MFC) use extracellular electron transfer (EET) to produce power from organic fuel. Contrarily, the production of electricity through photosynthesis using cyanobacteria requires only sunlight and water. Additional sources of organic carbon are not required. This offers tremendous potential for the development of a clean, renewable, and eco-friendly alternative energy source [11,12]. Cyanobacteria have the innate capacity to execute EET and have been proven to produce electricity in photo-bioelectrochemical (PBEC) fuel cells [13]. However, the power density attained with cyanobacteria was 0.035 Wm^2^ [12] is two orders of magnitude lower than that generated by exoelectrogens in MFC (7 Wm^2^ of electrode surface) [14]. In order to compete with biofuel cells and photovoltaic technology, the existing densities of cyanobacteria-based PBECs must be considerably increased. Genetic engineering may facilitate such a development [15].

In recent years, research in photosynthesis energy transfer utilizing cyanobacteria has increased significantly. Cyanobacteria have light-dependent electrogenic abilities in photo-bioelectrochemical cells that produce significant photocurrents. The genetic engineering of cyanobacteria to improve extracellular electron transport has been attempted, and it has been effective. Outer membrane cytochrome S (OmcS) is a non-native redox protein that was engineered to be expressed in *S. elongatus* PCC 7942 by genetic engineering. OmcS represents the most important factor in the metal-reducing ability of exoelectrogens such as *Geobacter* sp. The modified *S. elongatus* has more photocurrent production on a PBEC anode than the wild-type cyanobacterium [16]. The need for disposable sources of electricity contributed to the evolution of cyanobacterial biophotovoltaics to produce biophotoelectricity efficiently. Meanwhile, getting energy from cyanobacteria is challenging due to poor electron harvesting and extracellular electron transport. Biosynthesized gold nanoparticles are recommended because of their ability to act as an electrical conduit and as an intracellular light absorber, both of which improve the transit of photoexcited electrons across the cell membrane. Nanoparticles were generated both inside and outwardly on the membrane of *Synechocystis* sp. PCC 6803 by the bioelectrochemical reduction of metal ions. The power density was boosted by 33.6% using biophotovoltaics with intracellular gold nanoparticles [17].

Most of the biophotovoltaic research focuses on *Synechocystis* sp. PCC 6803 [18,19], *Synechococcus* sp. [20], *Nostoc* sp. [11,13]. *Synechococcus* sp. BDU 140432 has the greatest recorded power output at 610 mW m^−2^ [21]. The electrogenic activity of several wild-type cyanobacterial species and unspecified phototrophic consortia were compared by Pisciotta et al. [13]. Electrogenic production was highest in the microbial consortium from the freshwater pond and lowest in *Synechocystis* sp. PCC 6803. The photo-power output of two algae species, namely *Synechococcus* sp. WH 5701 and *Synechocystis* sp. PCC 6803 was investigated deeply [22]. *Synechococcus* showed the greatest biofilm-generating capabilities on ITO-PET anodes. Since it is a model organism in photosynthesis research with fully mapped genomes and also functions as a marker species for gene editing, *Synechocystis* sp. PCC 6803 continues to be the most favored cyanobacterium for biophotovoltaic studies [23].

## 5. The Effects of Nanoparticles on Genetically Engineered Cyanobacteria

**Figure 3:**
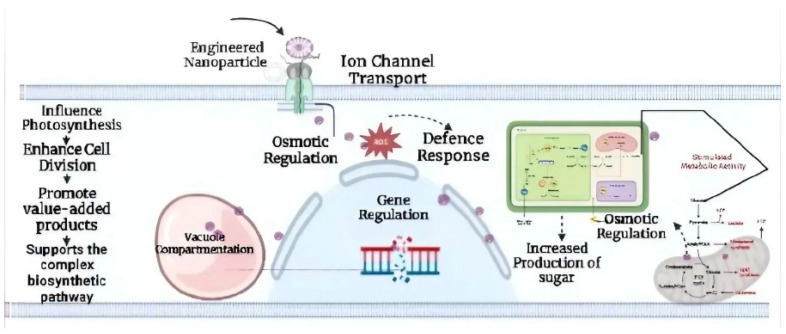
Employing engineered cyanobacteria for nanoparticle synthesis.

Poly-β-hydroxybutyrate is a carbon compound found in eco-friendly biodegradable polymers. *Synechocystis* sp. PCC 6803 was used to investigate the effect of genetic engineering and nanoparticle research on the increased production of polyhydroxybutyrate (PHB), a carbon component of biodegradable polymers [24]. PHB, a renewable and high-value-added carbon compound produced by CO_2_-fixing cyanobacteria, has been explored widely for its potential for excess production. *Synechocystis* species PCC 6803 is a variant of cyanobacteria capable of transforming and has had its whole genome analyzed, making it an ideal source for application in nanotechnology. Recombinant PHA negative mutant *Alcaligenes eutrophus* transformed with plasmids containing pha [exogenous phaECSyn or hybrid phaECAB (phaECSyn + phaABAe)] genes from *Synechocystis* sp. PCC 6803 and/or *A. eutrophus* grew effectively in nitrogen-depleted environments with sugar (gluconate, 0.5–2%) as the source of carbon [25]. In contrast, PCC 6803 generated 0.53 g/L of 3-hydroxybutyrate (3HB) under phosphate deprivation, and the byproducts were easily released out of the cells even without upregulation of transporter as it had endogenous phaAB and *E. coli* tesB (thioesterase) but not endogenous phaEC (phaEC genes deletion) [26]. The generation of PHB (and PHA) in cyanobacteria and other bacteria, both engineered and non-engineered, has also been investigated [27]. The amount of PHB biomass produced in terms of dry cell weight is quite high. But still, PHB overproduction has not been well studied using innovative expression vectors (Figure 3).

Polystyrene particles of micro and nano size are becoming a significant problem in marine ecosystems. The impact that polystyrene nanoparticles represent on freshwater producers is unclear. Researchers investigated the effects of short-term exposure to amino group-modified polystyrene nanoparticles (PS-NH2; 50 nm) on *S. elongatus* by analyzing its metabolite profiles and signaling pathways. PS-NH2 has an EC50 of 3.81 g mL^−1^ and is poisonous to *S. elongatus*. Analysis of non-targeted metabolites suggests that oxidative stress and membrane disintegration are responsible for PS-NH2 toxicity. In addition, the disruption of glutathione metabolism and the impairment of membrane integrity was verified using two modified strains of *S. elongatus*. These findings add to our knowledge of the potential effects of polystyrene nanoparticles on freshwater primary producers [28].

## 6. Genomic and Proteomic Insights on Cyanobacterial Heavy Metal Tolerance

The harmful effects of heavy metals (HMs) on aquatic and terrestrial life reflect a worldwide ecological threat. Effective cleanup of heavy metals from the ecosystem may restore soil fertility and ecological vitality. HM bioremediation may be possible with cyanobacteria because of their unique adaptations and powerful metabolic systems. Biosorption, bioaccumulation, activation of metal transporters, biotransformation, and stimulation of detoxifying enzymes are only a few of the mechanisms used by cyanobacteria to sequester and reduce the toxicity of heavy metals. Cyanobacterial cells bioremediate heavy metals by converting more toxic forms to less toxic forms (biotransformation), adsorbing heavy metals on cell surfaces, and absorbing and accumulating heavy metals inside the cells [29]. Deciphering the prospective genes and proteins that may be modified to increase the bioremediation effectiveness of cyanobacteria requires an appreciation for the physiological responses and control of adaption mechanisms at the molecular level. Some possible strain engineering targets include chaperones, cellular metabolites like extracellular polymers, biosurfactants, transcriptional regulators, metal transporters, phytochelatins, and metallothioneins [30]. *Nostoc* sp., *Anabaena* sp., *Phormidium* sp., *Oscillatoria* sp., and *Synechocystis* sp. have been identified from heavy metal-contaminated areas, suggesting their capacity to withstand HM stress [31] (Table 1).

Cyanobacteria use highly entrained gene expression to produce stress-responsive detoxifying proteins. Relative expressions of 278 genes, comprising metallothionein (smtA), cation efflux (catT, pacS), and ABC transporter, were observed in *Synechococcus* sp. IU 625 due to zinc stress [32]. *Synechocystis* sp. PCC 6803, lacking SmtA protein, contains ZiaA efflux pumps to remove excess Zn [33]. The thioredoxin (Trx) protein, which protects organisms from reducing stress, chelates HMs by a process similar to metallothioneins. *Trx* expression increases in response to Cd stress, but its chelating pathway in *Synechocystis* sp. PCC 6803 is unclear [34]. The cyanobacterial Trx binds to Cd ions and works as an HM sink to reduce Cd stress. Ferredoxin (Fed2–Fed9) expressed by cyanobacterial stress genes performs the main role during stress [35]. Fed1, Fed7, and Fed9 proteins engage in a ferredoxin-glutaredoxin-thioredoxin crosstalk pathway that reduces oxidative and mental stress. *Synechococcus* PCC 7002 and 7942 have 9 and 6 feeding genes, respectively [35]. The chaperones are essential for enduring environmental stress by refolding proteins and avoiding aggregations under denaturing stress [36]. In response to Cd, *Nostoc entophytum* ISC32 from hydrocarbon-contaminated soil upregulates GroEL and HptG chaperons [37]. The remarkable Cd absorption capacity of this strain may be attributable to its powerful antioxidant system and chaperons, corresponding with previously stated findings [38].

## 7. Cyanobacterial-Engineered Nanomaterials (NMs): A Heavy Metal Indicator

The earth contains naturally existing heavy metals, but human activities such as mining and processing, factory output, over-exploitation, and agriculture use of metals and their derived compounds have drastically expanded their presence in the natural surroundings [50]. The origin of cyanobacteria is connected with the geochemical history of metals. Some 2.7 billion years ago, the progression of cyanobacteria’s photosynthesis process coincided with large geochemical changes in iron diversification in aquatic systems [47,48,51,52]. As oxygen levels increased, soluble Fe (II) was oxidized to the insoluble Fe (III), significantly reducing iron concentrations in surface waters [53]. Cyanobacteria are now a major primary producer worldwide, as they can be found in every kind of aquatic environment that needs sufficient light for them to flourish. The nitrogen cycle is significantly influenced by cyanobacteria that fix atmospheric N_2_ and transform it into bioavailable forms of nitrogen (diazotrophs), in addition to their contribution to primary production [54,55]. However, hazardous cyanobacterial blooms pose a global danger to water supplies. In many ecosystems where cyanobacteria play a key role, iron is limited [56]. Iron is required roughly ten times more by cyanobacteria than by heterotrophic bacteria due to the existence of a photosynthetic electron transport chain [57]. Iron is required by diazotrophic cyanobacteria to support iron-rich enzymes that fix nitrogen [58]. The combination of a high iron requirement and poor iron bioavailability inhibits the primary production of cyanobacteria in several aquatic settings [56]. They may collect intracellular iron four to six orders of magnitude higher than other planktons. Carla Cherchi studied the impact of sublethal doses of nTiO_2_ on the nitrogen (N) metabolism of the nitrogen-fixing *Anabaena* PCC 7120 using the transcriptional level of biomarker genes involved in global N regulatory frameworks, N fixation, N assimilation, and N storage-specific pathways. The toxic response to light and dark exposure to nTiO_2_ was independently dictated by the circadian rhythms of cyanobacteria metabolism and the intrinsic characteristics of nTiO_2_ [59]. Such observations reveal that under nTiO_2_ environmental perturbation, cyanobacteria may alter their internal carbon and nitrogen homeostasis, impacting ecology food chain interaction. So, cyanobacteria must manage carbon and nitrogen metabolic processes and subcellular nutritional homeostasis to sustain production and adapt to altering conditions [19,43]. Cyanobacterial bioreporters were used to monitor ROS generation and free-ion release to evaluate the toxicity of metallic nanoparticles. Both the general toxicity bioreporter *Nostoc* CPB4337 [60] and the ROS-detecting *Nostoc* sp. were recombinant bioluminescent cyanobacterial strains [50]. *Photorhabdus luminescens* luxCDABE reporter genes linked to promoters of toxic, oxidative stress, and metal-sensitive genes make up the bioreporters. Biosensors for reactive oxygen species (ROS) have been studied in polluted river and industrial effluent environments. This underlines the use of cyanobacterial bioreporters as important environmental tools to assess the bioavailability, toxicity, and ROS production of metallic nanoparticles.

## 8. Aquaculture’s Next Frontier: Genetically Engineered Nanosized Cyanobacteria

The aquaculture industry is rapidly expanding to meet the rising consumer demand for seafood. New approaches are necessary to promote safe and effective fish production strategies and to develop environmentally friendly methods of protecting fish from disease outbreaks. Therefore, cyanobacteria, in combination with the broad range of genetic engineering approaches currently available, are seen as promising, ecologically benign, and highly sustainable microbial cell factories for the production of value-added products [61]. However, research on cyanobacterial EVs is still in its infancy, and many concerns remain. Extracellular vesicles (EVs) from *Synechocystis* sp. PCC6803 were investigated as a nanocarrier system for Zebrafish. *Synechocystis* EVs are biocompatible with fish larvae, causing minimal mortality or inflammatory responses. Researchers show that cyanobacteria can be engineered to synthesize recombinant proteins and load them into EVs. This research paves the way for the use of cyanobacterial EVs as a cutting-edge biotechnological tool in fish, with potential uses in transporting proteins and enzymes for modifying their nutritional status or inducing certain adaptive immunological responses [62]. *Synechococcus* sp. strain NKBG 15041c might serve as a potential, long-term source of ω3 fatty acids (ω3FA). In contrast to eukaryotic microalgae, with a more complex intracellular structure, cyanobacteria had simple intracellular partitions, with the cytoplasm, chloroplast, and endoplasmic reticulum housing the majority of the enzymes needed to catalyze ω3FA production [63,64,65]. The simplified intercellular organization of cyanobacteria makes it favorable for genetic engineering and nanotechnology [66] since designed proteins do not need to target particular intracellular organelles. Furthermore, cyanobacteria lack the rigid cell walls that certain eukaryotic microalgae possess, which might seriously affect fish guts and lower the survival rate in fish farming [67].

Antibiotic resistance genes (ARGs) are becoming more prevalent in aquatic ecosystems, causing severe environmental safety problems throughout the world. During the bloom condition, cyanobacteria had a much greater ARG. Extracellular DNA (eDNA) from cyanobacteria was shown to have a greater contribution and longer survival of ARGs than intracellular DNA (iDNA). The eDNA of cyanobacteria, which included the ARGs, was more stable at lower temperatures. *Microcystis* and *Synechococcus*, the two most common genus types in cyanobacterial blooms, have different ARG abundances. Planktonic cyanobacterial blooms are prevalent in eutrophic freshwater bodies and oligotrophic seas, where *Synechococcus* sp. accounts for 50% of biomass [68]. It has been proven that tailored cyanobacteria may serve as a prominent repository and source for the accumulation and distribution of ARGs in aquatic ecosystems [69]. Cyanobacteria isolated from Taihu Lake showed comparatively elevated levels of ARGs such as tetA, sulA, and sul1. In the warmer blooming phase, ARGs were found in larger concentrations and had better bacterial absorption efficiency in cyanobacterial strains, although ARGs in cyanobacterial eDNA were more stable at lower temperatures.

## 9. Genetically Engineered Cyanobacteria Nano-Formulation for High-Value Therapeutics

Cyanobacteria produce bioactive natural compounds with great economic and medicinal potential. However, only a few groups of them are produced in large quantities because of their low production and the expensive demand for isolating cyanobacteria species [70,71]. Scientists choose high-yielding strains and apply genetic engineering and nanotechnology to modify strains for value-added products [44]. Nanoparticles that directly modify or activate cell metabolism have been suggested and applied to highly impactful cyanobacteria [72]. Further efforts and strategies are required to create more suitable environments to produce effective therapeutics. Marine algae, in particular cyanobacterial species, are good resources for the synthesis of sulfated polysaccharides, which have a major interest in the field of nutraceutical, cosmetic industry, and drug development sectors [73]. In addition, polysaccharide-based nanoparticles are receiving a great deal of interest from nanotechnologists as innovative nanocarriers for cell imaging and therapeutic approaches [74] due to their high biocompatible, resilience, bio-degradability, cost-effectiveness, non-toxic character, and distinctive physiochemical characteristics. Since cyanobacteria-derived polysaccharides exhibit these characteristics, they hold great promise as biomaterials. Hence for this, they are of particular interest to the nanotechnology community. Moreover, several studies have looked at polysaccharide-based nanomaterials for use in medical fields such as antimicrobial activity, drug delivery, gene transfer, cancer therapy, and wound healing [75,76]. Polysaccharides produced by cyanobacteria have antiviral and antioxidant properties. Cyanobacterial polysaccharides produced by *Arthrospira platensis* have antiviral properties in vitro and in vivo against vaccinia and the ectromelia virus. Nostoflan, an acidic polysaccharide from *N. flagelliforme*, has antiviral properties against enveloped viruses like influenza [77]. Antioxidative molecules such as sulfated polysaccharides from cyanobacteria may prevent cancer in humans. Polysaccharides derived from *Spirulina* sp. exhibit pharmacological properties. *Spirulina* sp. polymers inhibit tumor cell growth in vitro and in vivo. Cyanobacteria components could be more beneficial when combined with nanomaterial [78,79] (Table 2).

Identification of a natural oxadiazine nocuolin A (NoA) from several genera with a specific 1,2,3-oxadiazine composition and representing the subsets of the ostensible genes for the synthesis of NoA in their genome confirms that these cyanobacterial genera have a distinct biodiversity of natural products [80]. NoA-induced cell death was claimed to share the same characteristics as caspase-dependent apoptosis. NoA was reported to have anti-proliferative properties on a plethora of cancerous cells, particularly p53-mutated cell lines, with an IC50 value ranging from 0.7 to 4.5 M [79]. NoA represents a new class of heterocyclic natural metabolites. Comparable heterocyclic arrangements have only been found in synthetic compounds, primarily 1,3,4- or 1,2,4-oxadiazines, where the N–N–O system is curtailed by a carbon atom [81]. N–N–O linkages were only reported in 1,2,3-oxadiazole derivatives [82]. Whereas an open ring shape is more stable, as anticipated by quantum chemical modeling, it was first thought that the occurrence of a closed 1,2,3-oxadiazole ring seemed improbable [79]. As shown in several synthetic 1,2,3-oxadiazole derivatives, the steady state may be altered to favor the cyclic form by adding the right substituents [83]. The exploration of NoA provides new perspectives on the chemistry and occurrence of heterocyclic compounds with N-N-O linkages. The 1,3,4-oxadiazoles, in particular, provide a significant platform for therapeutic discovery from a pharmacological standpoint [81]. There is significant interest in creating novel 1,3,4-oxadiazole compounds as it has been shown that oxadiazole structures display a wide spectrum of biological activities. Submicromolar concentrations of synthetic 1,3,4-oxadiazine compounds were highly effective on cancerous cells [84].

## 10. Cyanobacterial Cell Factories—A Plastic Scavengers

One of the most frequently researched and prominent concerns of the 21st century is plastic pollution created by non-biodegradable polymers. The rapid proliferation of single-use plastics has made this kind of pollution a major problem worldwide. For this reason, research on biodegradable polymers like polylactic acid (PLA), polyhydroxybutyrate (PHB), and poly-caprolactone (PCL) has gained greater attention [110]. Most experts agree that creating biodegradable polymers like polylactic acid (PLA) is the best long-term solution. The current PLA manufacturing technique depends significantly on agri-producible substances like rice bran, and maize, leading to rivalry for resources between raw materials and crop production. Hence, scientists used metabolic engineering and high-density culture (HDC) to turn cyanobacteria into a cell factory for the in vivo biosynthesis of PLA directly from CO_2_. The strain of *Synechococcus elongatus* PCC7942 was engineered to generate D-lactic dehydrogenase, propionate CoA-transferase, and polyhydroxyalkanoate synthase. In addition, when bioengineering techniques were used consistently, including promoter tuning, acetyl-CoA self-regulation, and carbon-flux redirection, PLA output virtually quadrupled compared to the control. In vivo, PHA granules are amorphous and deformable [111]. Based on a comprehensive review of the most current research, the synthesis of PHA biopolyesters under regulated circumstances takes place in bioreactors with a variety of geometries like stirred tank bioreactors, cylindrical bioreactors, airlift bioreactors and is based on many culture schedules, including batch, fed-batch, and continuous cultivation [112]. Researchers from various fields, notably systems biology, computational biology, genetic engineering, enzymology, polymer chemistry, and process engineering, are involved in the multidisciplinary study of PHA biopolyesters [112] (Table 3).

*Synechocystis* PC 6803 transformation has been used in many studies to examine their capacity to make PHB, biohydrogen, isoprene, and other chemical products [113,114]. Over-expression of native PHB genes increased PHB output from 10% to 26% in *Synechocystis* PCC6803 [115]. *Synechocystis* PCC 6803 was genetically modified to produce the PHB monomer hydroxybutyrate (both (S)- and (R)-3-hydroxybutyrate (3HB)) by inactivating PHB polymerase [25]. In addition, two more approaches for synthesizing monomers from acetyl-CoA were suggested. Without altering the enzymatic activity of the transporters, the monomers were easily secreted, and titers of 533.4 mg/L 3HB were achieved. Recent genetic engineering of *Synechocystis* PCC 6803 focused on central carbon metabolism rather than over-expressing or adding PHB synthase genes [116]. The production of a recombinant phosphoketolase (XfpK) from *Bifidobacterium breve* achieved 12% PHB yield, titers of 232 mg/L, and productivity of 7.3 mg/L/day, which has been claimed to be the maximum yield. *C. reinhardtii* and *P. tricornutum* were genetically engineered to generate PHA biosynthesis genes and yielded 6 × 10^−4^% and 10.6% PHB, respectively [117]. These studies might help in screening eukaryotic algae with high Acetyl-CoA pools for PHB production genes. A 10% yield was achieved without plastid targeting, codon optimization, nuclear integration, or a non-inducible promoter. Suppressing glycogen or other lipid metabolite synthesis pathways increases carbon transport to PHB. Creating a PHB de-polymerase knockout cyanobacteria that can’t metabolize intracellular PHB might boost yield and production [118].

## 11. Crop Improvement Using Cyanobacteria-Mediated Nanoparticle Gene Delivery

Agriculture serves to be the primary source of income, livelihood, and wealth in developing nations, engaging around 50% and 90% of the population. Human activities may lead to soil deterioration, salinity, and sterility, which may reduce the amount of fertile land and adversely affect agricultural productivity [128]. In agriculture, the use of cyanobacterial NPs to improve food production and disease eradication is still in its nascent stage. Moreover, there is little or no evidence of the practical usage of engineered NPs (ENMs) in agriculture [129]. Nano-biotechnology may revolutionize agriculture and food with novel methods for disease prevention and control, plant nutrient absorption, slow-release fertilizers, and environmental safeguards. Natural biofertilizers like cyanobacteria may improve agricultural and ecologically sustainable responses differently [130]. An estimated one billion hectares (ha) of salinized soils may be restored using biological, physical, and chemical remediation techniques using genetically engineered cyanobacterial species. Engineered *Nostoc* sp. and *Anabaena* sp. are particularly well-suited to combat the soil salinization condition by fixing atmospheric nitrogen and creating an extracellular matrix and suitable solutes [128]. Soil salinity, weed development, and weed decomposition can all be slowed with the aid of cyanobacteria.

Cyanobacteria have the special capability to fix nitrogen in rice paddies, which increases the yield and growth of paddy. Their nitrogen-fixing abilities and other beneficial impacts on soils and plants give them a prominent position in agricultural contexts, including rice farming. Major nitrogen-fixing organisms include *Anabaena*, *Aulosira*, *Nostoc*, *Calothrix*, *Plectonema*, etc. These species act as biofertilizers, enabling atmospheric dinitrogen to be converted to ammonium, solubilizing fixed nutrients, and turning insoluble phosphorus in the soil into phytoavailable forms [131]. The latest research found that radish seedlings have higher chlorophyll and elements content after being treated with *Spirulina platensis* supernatants. These results demonstrated the feasibility of using cyanobacterial extract in modern agriculture and horticulture. Spirulina supplements might promote sustainable agriculture, assuring food supply to satisfy growing populations and environmental goals [132]. Cyanobacteria perform a significant function in the soil microbial community and aid in reversing the soil degradation impact [133]. Fertilizer made from cyanobacteria and microalgal biomass is used to revitalize depleted soil by increasing their water binding ability and nutrient content [134]. Overall, biofertilizers like cyanobacteria are a more ecologically and economically sustainable solution than artificial fertilizers like urea since they need less capital and energy. On the contrary, researchers must consider the exposure of cyanotoxins in agricultural soils owing to the recovery of a large amount of cyanobacterial biomass from water and distribution to farmland without pretreatment.

## 12. Cyanobacteria and Nano-Sand-Stabilized Biocrust

Humans encounter environmental challenges, including desertification and eutrophication. Cyanobacteria biocrusts are an efficient way to prevent desertification [135]. The formation of biocrust by cyanobacteria inoculation is regarded as a significant and promising bioengineering strategy for mitigating desertification. Furthermore, because of the harsh biological conditions of the desert, it is difficult for permanent biocrust to develop fast on the sand surface. A nano-sand-stabilizer network structured nanocomposite was developed using attapulgite and carboxymethyl cellulose [136]. Cyanobacterial biocrust has been successfully used in desert fixing as a potential eco-friendly technology [88,137,138]. Over the last two decades, cyanobacteria have been inoculated either alone, in combination with other cyanobacteria, or with plants or a straw-checkered boundary [139,140,141]. Furthermore, it was difficult to rapidly establish a stable microenvironment for cyanobacteria to settle down using these approaches, which made it unfavorable for cyanobacteria to construct biocrust. Recent research has focused on the use of chemical methods to improve biocrust formation efficiency. One such method is the use of modified water-borne polyurethane and cationic poly copolymer emulsion [142,143,144]. The combination of filamentous cyanobacteria and fly ash might improve surface soil stability owing to their cumulative impact [145]. Sodium alginate was applied to enhance cyanobacterial colonization, growth, and the production of biocrust on sand surfaces [146]. One of the current research areas in the field of ecosystem reclamation is the coupling of mixed cyanobacteria (MC) with nanocomposites to generate biocrust. Desert biocrust production is limited by a lack of plantlets and nutrients. Eutrophic water that contains aquatic cyanobacteria (AC), nitrogen, and phosphorus may be a low-cost source of plantlets and nutrients. In recent decades, scientists synthesized a networked nanocomposite using a metal-organic framework (MOF) and carboxymethyl cellulose (CMC). Network-structured nanocomposites with a large specific surface area and various surface groups showed excellent water and nutrient retention [135].

## 13. Application of Cyanobacteria in Carbon Capture

The sustainable synthesis of fuels and hydrocarbons from renewable resources is essential due to rising petroleum usage and increasing carbon emissions. Lactate, 1,2-propanediol, isobutyraldehyde, and butanol are some of the compounds and fuels that can be formed by genetically modifying cyanobacteria [147,148]. This highlights the need to develop engineered cyanobacteria for the production of intermediates, which can be further processed into a wide variety of useful products using widely accepted biological and chemical pathways. The above approach could avoid possibly difficult and time-consuming genetic manipulation in cyanobacteria and make the most of well-known biosynthesis processes and microbial producers, particularly if recombinant pathways are irreconcilable with cyanobacterial metabolic activities or the products are highly toxic to cyanobacteria. The engineered cyanobacterium that generates intermediate may be used in bio-fermentation and hydrocarbon production. Therefore, a novel approach is shown for the photosynthetic generation of C3 platform compounds from CO_2_ employing a tailored *S. elongatus* PCC7942 as the kernel. An elevated generation of the intermediate from CO_2_ was needed to function better as a flexible cyanobacterial kernel. Expanding heterologous routes, co-cultivating cyanobacteria and glycerol utilizers, fermenting glycerol, or chemical synthesis are all viable options for using the cyanobacterial kernel in the manufacture of chemicals and fuels. Important C3 platform compounds like docosahexaenoic acid, 3-hydroxypentanoic acid, and 1,3-pyrrolidone were produced from carbon dioxide utilizing the kernel. This proved the promise of the novel technique for manufacturing chemicals and fuels from CO_2_ in an environmentally friendly manner [149].

Global warming and the energy crisis have made the use of microalgae in photobioreactors for CO_2_ bio-mitigation and biomass yield enticing [150]. Using modified Carbonic anhydrase (CA)-producing cyanobacterial strains, CO_2_ sequestration and biomass production were measured. An improved photobioreactor-based microalgal CO_2_ mitigation system by integrating chemical and biological CO_2_ fixation was developed. The cyanobacteria were genetically modified to synthesize and secrete CAs in the medium [151]. CAs effectively converted dissolved CO_2_ to HCO_3_. In addition, the cyanobacteria absorbed HCO_3_, which was subsequently converted into biomass by photosynthesis. It is proved that CO_2_ can be sequestered sustainably by combining chemical and biological CO_2_ fixation in a microalgal photobioreactor system.

## 14. Genetically Improved Cyanobacteria for Biofuel Production

In recent years, cyanobacteria have been identified as potential microbial substrates [152]. Photosynthetic microorganisms create the biofuels of the third generation. By employing genetic engineering approaches, it is possible to generate cyanobacteria-based biofuel production with high conversion rates and cheap costs [153]. Like most cases, to produce the necessary high-value chemical or next-generation biofuel, cyanobacteria strains must be genetically modified using foreign genes. This could entail integrating new genes or new pathways into the species based on the desired outcome. The Calvin-Benson Cycle (CBC), found in cyanobacteria, has been responsible for CO_2_ fixation [154]. CBC produces pyruvate, an intermediary in biosynthetic pathways like methylerythritol phosphate (MEP) and mevalonate (MVA). Dimethylallyl pyrophosphate and Isopentenyl pyrophosphate, which are fundamental precursors to terpenoids, are produced through MEP and MVA processes. MEP is inherent in cyanobacteria, whereas MVA was added by genetic engineering. The MVA method improves carbon flow to selected cyanobacterial products through the MEP pathway [155]. Most of the cyanobacterial species have now been sequenced and are accessible for usage at genome databases. Thirty-nine strains of cyanobacteria are included in the CyanoBase genome database. The number of cyanobacterial genome sequences is increasing and now exceeds 281 [156].

The recovery of lipids from microalgae biomass and the esterification of diesel is the most frequent use [157]. Genetically modified cyanobacteria that produce and secrete ethanol and butanol are recently reported [158,159]. However, the cyanobacteria-based approach is thought to be preferable because it is flexible to molecular engineering techniques [160]. Although life cycle assessment (LCA) has become the standard method of environmental evaluation due to its many advantages, it might be challenging to adapt to new technologies (ex-ante) on cyanobacteria to derive biofuels. Two life cycle assessment (LCA) studies for ethanol generation using cyanobacteria have been conducted, with slightly differing results [161]. Evaluating the environmental implications and cumulative energy demand of cyanobacteria-produced n-butanol using cradle-to-grave consequential LCA proved that a designed cyanobacteria-based system will improve productivity [162].

### 14.1. Benefits

1. Non-toxic to the surrounding environment.
2. Extremely efficient with regard to cost.
3. Does not result in the production of any undesirable residues.
4. Reduces transportation costs and may be performed immediately on-site.
5. May be used in conjunction with several other therapeutic methods.

### 14.2. Challenges

1. Some compounds are partially degradable.
2. The outcomes that were seen in the laboratory may turn out differently when the experiment is carried out in the field.
3. There is a possibility that the hydrological and geochemical parameters of the site may change over time.
4. Water and fertilizers are other issues for large-scale cyanobacterial cultivation.
5. Rapidly changing climatic conditions and pollution threaten the economic feasibility of outdoor cyanobacteria farming.

## 15. Genetically Modified Cyanobacteria and the Threats They Entail

The environmental risk of commercial production must be evaluated, as is the case with all Genetically Modified Organisms (GMOs), and suitable countermeasures must be implemented. An earlier risk assessment for genetically modified microalgae indicated that hazards to human health, the environment, and the economy were minimal but not zero [163]. Since genetically modified cyanobacteria can be grown outside, possibly in open ponds, they found that these cells have a higher chance of getting into the environment than cells grown in a typical industrial setting. Any escaped genetically engineered cyanobacteria would be in direct competition with natural species for food sources. In doing so, they may alter the ecology of biological communities while continuing to produce the useful products for which they were developed. They might have low or moderate toxicity to other species depending on the substance. The altered cyanobacteria might become a food source for other creatures, with potentially unforeseen consequences for the ecosystem. There is concern that genetically engineered (GE) cyanobacteria might exacerbate eutrophication, which can lead to a decrease in dissolved oxygen after an algal bloom. Horizontal gene transfer from GE cyanobacteria to other organisms might transmit antibiotic-resistance genes, as these specific genes are routinely utilized as selection markers. A proprietary cyanobacteria species named AB1 was modified by Algenol to make ethanol [164]. Algenol received a judgment from the USDA Biotechnology Regulatory Services-APHIS in 2009 that the strains they used were not harmful to plants, animals, or people and so were not protected under the Plant Protection Act. However, under APHIS, interstate trafficking of hybrid algae still required a permit. Algenol’s indoor R&D was exempt from TSCA permits (TSCA). In cyanobacteria, a plethora of deadly genes have been used for biocontainment as well as for counterselection, including phage lysis proteins and proteins from the toxin-antitoxin system [165]. The antitoxin may be co-expressed at a low level to avoid leaky production of the toxic protein from decreasing growth rates in the lab, which is a major advantage of employing toxins from antitoxin systems. Cyanobacteria cultivation commonly uses phosphate as the phosphorous source. The genes involved in phosphite transport and phosphite oxidation to phosphate are known. Thus, they may be used by some species. Since not all organisms can utilize phosphite, introducing these genes into cyanobacteria and growing them on a medium lacking phosphate has been proven to be beneficial in minimizing biological contamination [166]. Genotypes developed from cyanobacteria were auxotrophic for phosphite and either melamine or urea as nitrogen sources. *Synechococcus* sp. PCC7942 was engineered to be a phosphite auxotroph by replacing its native phosphate transporters with those from *Pseudomonas stutzeri* WM88, which also provided the genes for phosphite transport (HtxBCDE) and phosphite oxidation (ptxD) [167]. After 28 days, the researchers observed that the escape frequency was below the 3.6 1011 per colony forming unit detection threshold. The modified strain did not thrive on BG11 medium made with sterilized fresh water from a natural source, despite the possibility that this water contains trace amounts of phosphite (with or without phosphate). Growing the modified strain alongside the wild-type *Synechocystis* sp. PCC6803 allowed for an evaluation of the potential for horizontal gene transfer. Placing the mixed culture on the kanamycin-selective medium inhibited the phosphate transporter. Each of the 28 screened colonies tested positive for *Synechococcus elongatus* 7942, as opposed to the wild-type *Synechococcus elongatus* 6803 [168]. A recent study demonstrates that ammonium transporters may be knocked off to create synthetic auxotropies of nitrogen sources. In most cases, the cyanobacteria are endowed with the capability to fix atmospheric N2, degrade organic wastes and residues, remove toxins and heavy metals, pesticides, and other xenobiotics, catalyze nutrient cycling, suppress the growth of pathogens in soil and water, and generate few active molecules. On the one hand, humans face unprecedented threats to human health and the environment, while on the other, researchers have chances to alter the way things are accomplished. The regulations governing the use of genetically modified cyanobacteria need a wider decision-making foundation. The post-release effects of genetically modified cyanobacteria may be mitigated by using preventative and precautionary measures based on risk assessment and management.

## 16. Conclusions

In recent decades, genetically engineered cyanobacteria-mediated nanoparticle synthesis has shown great promise for the improvement of innovative methods, as it permits the environmentally friendly, energy-efficient, and cost-effective synthesis of nanoparticles of varying forms and sizes. Genetically modified cyanobacteria-mediated nanoparticles have unique physical, chemical, and biological properties that have anticancer, antibacterial, and photocatalytic applications. Recent remarkable achievements in the area of synthetic biology of cyanobacteria and nanomaterial synthesis from the species have provided researchers hope for the widespread implementation of this breakthrough in the field of industrial applications. This review provides the basic knowledge required to create a powerful system for using cyanobacteria in a wide range of settings, including environmental impacts, sustainable agriculture practices, therapeutic procedures, biofuels, and other relevant byproducts. In considering their unique features and evolving diversity, cyanobacteria might be a rich source of natural commodities. Based on these findings, researchers, scientists, and industrialists should engage in establishing the framework for further study of cyanobacterial innovation since these microorganisms have the potential to be used in a wide range of applications.

## Figures and Tables

**Table 1:** Cyanobacterial species used for the removal of different heavy metals.

S.No	Species	Metal	Incubation	pH	Temperature	Bioremediation Value	Ref.
1	*Synechococcus* sp. and *Aphanocapsa* sp.	Chromium (Cr), lead (Pb)	240 h	7.8	27 °C	Cr^−^(63.8 to 56.2 μg/L), Pb^−^ (418 to 239 μg/L)	[39]
2	*Nostoc muscorum*	Cu(II), Zn(II), Pb(II) and Cd(II)	72-h	8.0	25–30 °C	Pb (II) (96.3 %) and Cu (II) (96.42 %) were the most abundant, closely by Cd(II) (80.04 %) and Zn(II) (71.3 %).	[40]
3	*Phormidium bohneri*	NO_3_^−^, PO_4_^3−^	14 h light–10 h dark.	_	15 °C	80 to 350 μmol photon m^−2^ s^−1.^	[41]
4	*Phormidium laminosum*	Arsenic,	24 h	7.8	_	37.17 μg g^−1^	[42]
5	*Nostoc sphaeroides*	Cr^3+^, Pb^2+^	4 h	5.0	25 °C	116.28 and 22.37 mg g^−1^	[43]
6	*Arthrospira platensis*	Cu^2+^ and Ni^2+^	48 h	5.0–6.0	30 ± 2 °C	Cu^2+^-(2.33–3.08 mg/g), Ni^2+^- (2.14–2.84 mg/g).	[44]
7	*Spirulina* sp.	Cr^3+^, Cd^2+^, Cu^2+^	100 h	7.0	35 °C	Cr^3+^-(185 mg g^−1^), Cu^2+^-(196 mg g^−1^), Cd^2+^-(159 mg g^−1^)	[45,46]
8	*Anabaena torulosa*	Cd^2+^	4–5 h	7.0	18.5Â °C	8 mg L^−1^	[47,48]
9	*Synechocysis* sp. PCC6803	Arsenite	72 h	6.2	25 °C/20 °C day/night	0.9 and 1.0 mg kg^−1^ DW	[49]

**Table 2:** Bioactive compounds from cyanobacteria and their potential use in therapeutics.

S.No	Strains	Bioactive Compounds	Structure	Potential Activities	Reference
1	*Spirulina*	c-phycocyanin	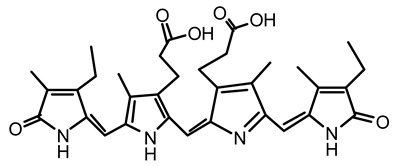	Neuroprotective action through cyclooxygenase 2 inhibition (COX-2)	[85,86]
2	*S. platensis* (synonym *Arthrospira platensis*)	Gamma linolenic acid (GLA)	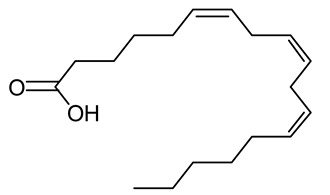	Reduces blood pressure and improves lipid metabolism	[87]
3	*Lyngbya* sp.	Apratoxin A	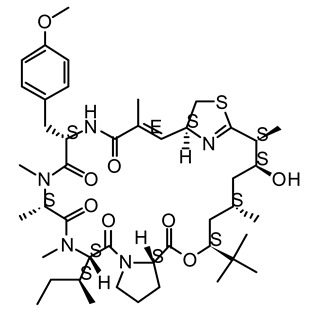	Inhibits the JAK/STAT signaling pathway by downregulating IL-6 signal transducer (gp130)	[88]
4	*Lyngbya* sp.	Bisebromoamide	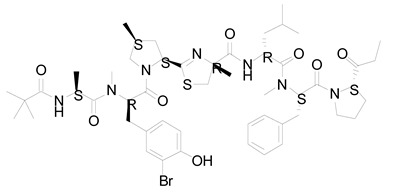	Actin stabilizing property	[89]
5	*Lyngbya* sp.	Biselyngbyaside	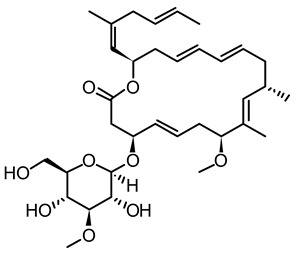	Cytotoxic/anti-proliferative	[90]
6	*Geitlerinema* sp.	Ankaraholide A	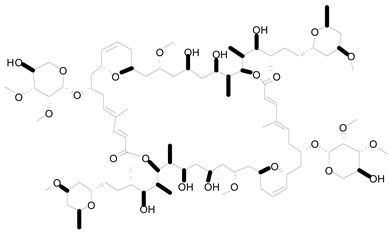	Loss of F-actin	[91]
7	*Dolabella auricularia*	Aurilide	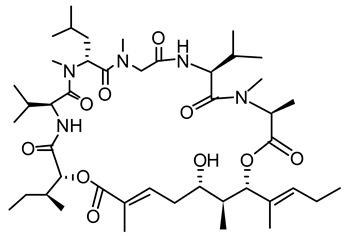	It increases mitochondrial-induced apoptosis by binding PHB1 and initiating OA1 proteolysis (OPA1)	[92]
8	*Nostoc linckia* and *N. spongiaeforme* var. tenue	Borophycin	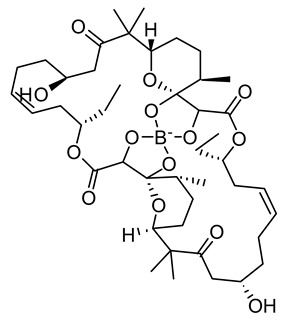	Cytotoxic against colorectal cancer	[93]
9	*Calothrix* sp.	Calothrixins A	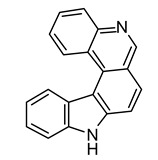	Triggered apoptosis and G2/M cell cycle arrest in all cancer cell lines	[94,95]
10	*Symploca* sp.	Carmaphycins A and B	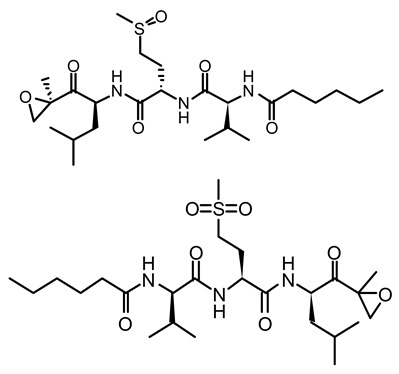	High levels of activity against proteasomes	[96,97]
11	*Lyngbya majuscule* and *Phormidium* sp.	Caylobolide A and B	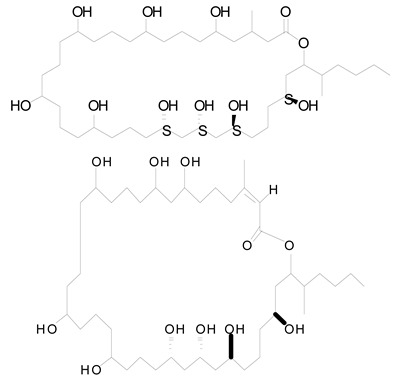	Produce cytotoxicity in cancer cells. Caylobolide A fights HCT-116 colon cancer	[98,99]
12	*Leptolyngbya* sp.	Coibamide A	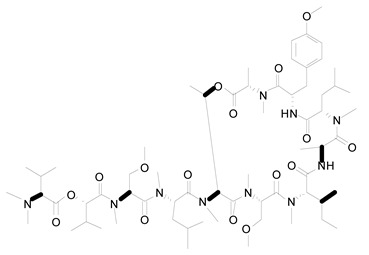	mTOR-independent autophagy and glioblastoma cell death	[100]
13	*Nostoc* sp. var. ATCC 53789	Cryptophycin	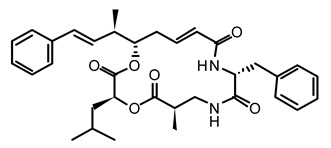	Suppress microtubule synthesis; anti-tumorigenic	[101]
14	*Lyngbya majuscula*	Desmethoxymajusculamide C	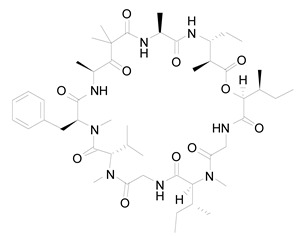	Depolymerizes actin cytoskeleton, disrupting cell microfibril network	[102]
15	*Dolabella auricularia*	Dolastatin	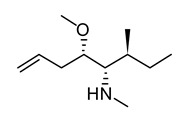	Interference with microtubule assembly arrests the cell cycle in G2/M, causing apoptosis	[103]
16	*Symploca* sp. VP64	Symplostatin 3	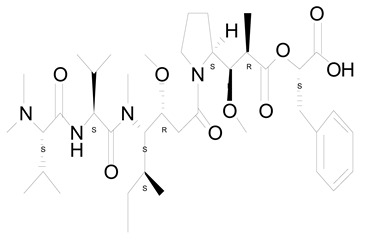	Microtubules disruption	[104]
17	*Lyngbya confervoides*	Grassypeptolide	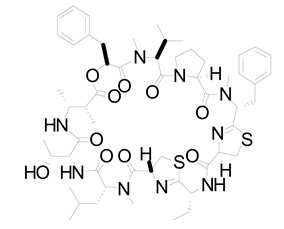	G1 cell cycle arrest	[105,106]
18	*L. majuscula*	Hectochlorin	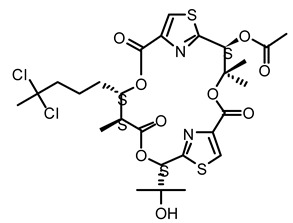	Increases actin polymerization	[107]
19	*Symploca* sp	Hoiamide D	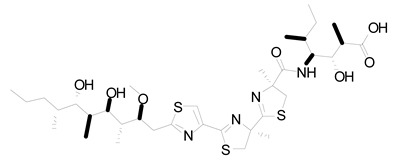	Blocking p53 and high-fidelity minichromosome 2 (p53/HDM2)	[108]
20	*Oscillatoria margaritifera*	Veraguamides A	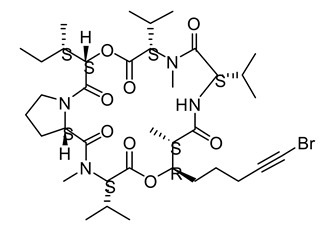	Cytotoxic effect	[109]

**Table 3:** Production of PHB by cyanobacteria.

S.No	Species	Approach/Analysis	Culture Condition	Source	Biomass Conc.	Ref.
1	*Synechocystis* sp. PCC 6803	Systems Metabolic Engineering	Photoautotrophic	Glucose	1.37 gDW/L	[119]
2	*Anabaena* sp.	HPLC and FTIR	Photoautotrophic	Fresh Water, Marine water	2.314 ± 0.012 mg/L	[120]
3	*N. muscorum* NCCU-442	FTIR, NMR, and GC MS	Heteroautotrophic	Glucose, maltose, fructose, sucrose, lactose and starch	Accumulation of 26.37% PHB	[121]
4	*Aulosira fertilissima*	Composite rotary design (CCRD)-statistical package, Pyris Diamond Differential Scanning Calorimeter	Mixotrophy (Chemoheterotrophic and photoautotrophic)	Sucrose, fructose, glucose, maltose	1.59 g L^−1^	[122]
5	*Calothrix scytonemicola* TISTR 8095	HPLC, NMR	Photoautotrophic	Atmospheric carbon dioxide (CO_2_)	25.2% (*w*/*w* DW	[118]
6	*Nostoc muscorum*	Spectro- Photometer	Photoautotrophic	Acetate, glucose, maltose, fructose, and ethane	8.6%, *w*/*w*, of the dry cell	[123]
7	*Arthrospira platensis RRGK*	FTIR, DSC, TGA, and XRD	Photoautotrophic	Sodium bicarbonate	1.101 g L^−1^	[124]
8	*Cyanobacterium Spirulina LEB 18*	Photobioreactor	Mixotrophic	Glucose or sodium acetate	44.2	[125]
9	*Spirulina subsalsa*	IR, NMR, TGA, and DS	Phototrophic	Atmospheric carbon dioxide (CO_2_)	1.97 g L^−1^	[126]
10	*Synechocystis* sp. PCC 6803	Propanolysis method	Photoautotrophic	Glucose, maltose, fructose	38	[127]

## Data Availability

Not applicable.
